# Progressive Cerebral Small Vessel Disease Caused by an Autoimmune Response to Intravesical Bacille-Calmette-Guérin Treatment

**DOI:** 10.3389/fneur.2020.484282

**Published:** 2020-10-26

**Authors:** Anouck Becker, Iris Quasar Grunwald, Marcus M. Unger, Stefanie Behnke, Joerg Spiegel, Umut Yilmaz, Silke Walter, Klaus Faßbender

**Affiliations:** ^1^Department of Neurology, Saarland University, Homburg, Germany; ^2^Neuroscience Unit, Faculty of Medicine, Anglia Ruskin University, Chelmsford, United Kingdom; ^3^Radiology Department, Southend University Hospital National Health Service Trust, Essex, United Kingdom; ^4^Department of Neuroradiology, Saarland University, Homburg, Germany

**Keywords:** cerebral vasculitis, small vessel disease, BCG, Bacille-Calmette-Guerin (BCG), autoimmune disease, urothelioma, molecular mimicry

## Abstract

Systemic BCGitis and autoimmune diseases are possible adverse events of intravesical Bacille Calmette-Guérin-(BCG)-instillations in the treatment of urothelioma cancer. We present the case of an 83-years-old male patient with rapid progressive symptoms of dementia up to mutism as well as tonic seizures. Immune-mediated cerebral small vessel disease was diagnosed and retraced to former instillations of BCG. Intense immunosuppressive treatment was performed and clinical restoration was achieved within several months. While cerebral vasculitis due to BCGitis has already been described before, this is to our knowledge the first case report to illustrate an immune-mediated small vessel disease after BCG-instillations. This should be considered in patients with rapidly progressive dementia-like symptoms treated with BCG, as an immunosuppressive treatment can be highly effective even at severe clinical stages.

## Case report

An 83-year-old male patient with rapid progressive symptoms of dementia up to mutism, recurrent tonic seizures, and gait impairment up to immobility was transferred to our clinic.

Magnetic resonance imaging (MRI) of the brain, cerebrospinal fluid (CSF), and blood analysis including paraneoplastic antibodies had been negative beforehand. Treatment with levetiracetam had been started. Additional musculoskeletal pain was diagnosed as polymyalgia rheumatica, confirmed by positron emission tomography (PET) imaging, and treated with 20 mg prednisolone daily.

Medical history included bladder urothelial carcinoma, treated with intravesical *Bacille Calmette-Guérin* (BCG)-instillations from 2012 to 2017, successfully treated caecum carcinoma, mild arterial hypertension, and a transient ischemic attack.

**Figure 1:**
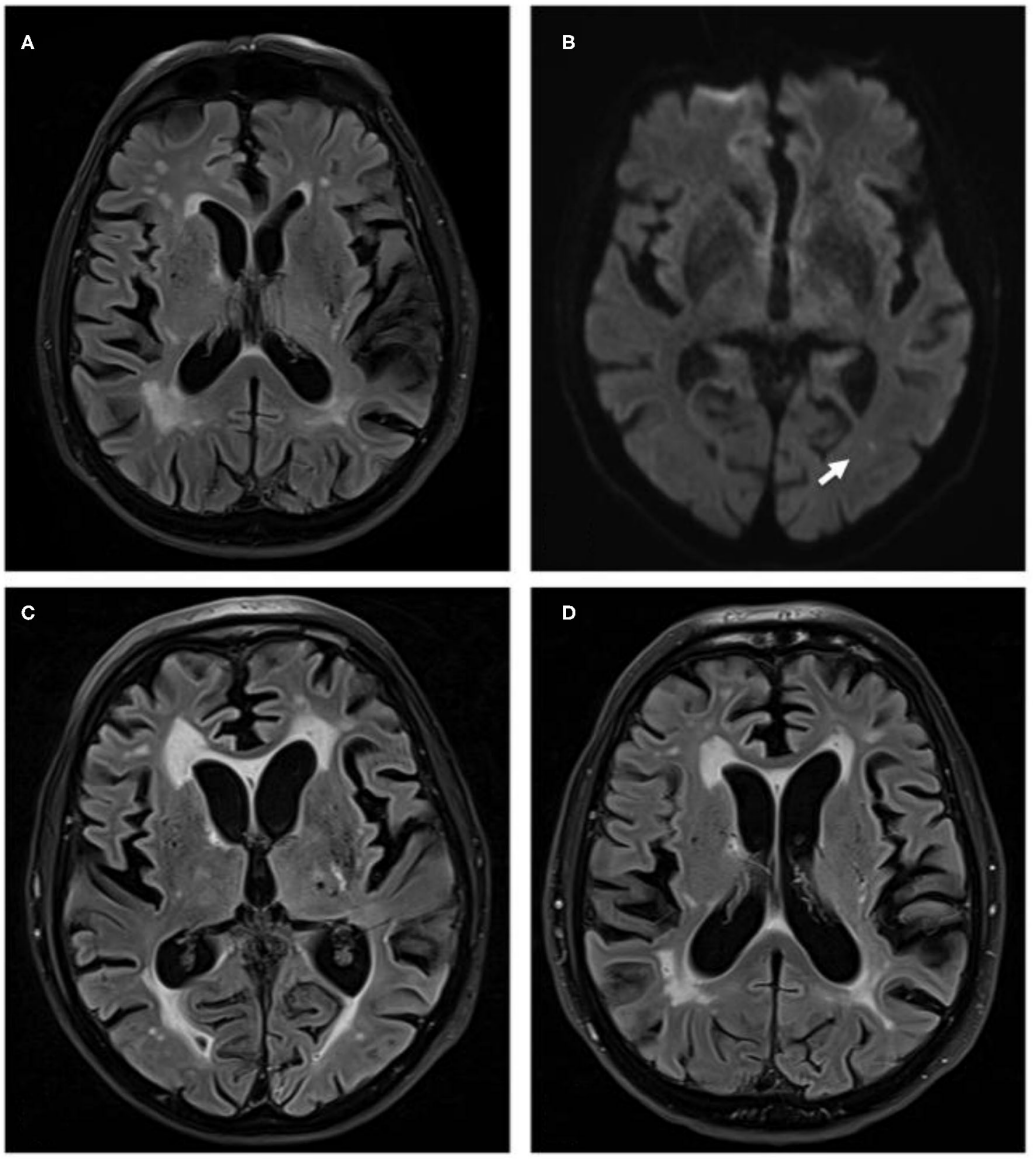
**(A)** Magnetic resonance brain images with fluid attenuated inversion recovery (FLAIR) sequences of October 2017, when patient presented with recurrent seizures. Images show distinct cerebral small vessel disease. **(B)** Diffusion weighted imaging sequences of the same examination show point-shaped infarction in left-sided MCA-territory (white arrow). **(C)** FLAIR sequence of April 2018 when patient deteriorated clinically with clear increase of white matter damage. **(D)** FLAIR sequence of October 2018, when patient presented for fifth cycle of cyclophosphamide treatment and with a marked clinical improvement. Images show a clear reduction of small vessel disease.

Cerebral MRI showed severe small vessel disease and a fresh ischemic lesion in the left middle cerebral artery territory (Figures 1A,B). Repeated lumbar puncture was normal. EEG showed deceleration with left-sided accentuation, but no signs of epileptic activity. We changed levetiracetam to valproate and Phenobarbital, resulting in successful control of seizures. Active polymyalgia rheumatica was confirmed and oral prednisolone treatment was increased to 30 mg daily. The patient was discharged being able to communicate normally and walk with aids.

He represented again 2 months later with elevated liver enzymes, presumably caused by combination of anti-epileptic valproate and chloroquine medication, which had been started as additional treatment of polymyalgia rheumatica in the meantime. Medication was changed to lamotrigine and prednisolone was again increased to 20 mg daily after stopping chloroquine. Liver enzymes normalized within 11 weeks.

Three months later, the patient was again admitted because of a dramatic clinical deterioration. He was somnolent, only spoke some words and followed simple commands, no motor deficit, but unable to walk with flapping tremor at limbs and trunk. The working diagnosis was a valproate-induced encephalopathy. We accelerated the change from valproate to lamotrigine treatment, but without clinical improvement. The brain MRI showed progressive small vessel disease (Figure 1C). Neither CSF analysis including dementia marker nor autoimmune serum antibodies for limbic encephalitis were positive. EEG examinations again showed no epileptic abnormalities. Additional chest X-ray, colonoscopy, gastroscopy, whole-body fluorodeoxyglucose PET imaging to exclude seronegative paraneoplastic disease, rectal biopsy to exclude amyloidosis were negative.

The patient's clinical state worsened with necessity of intensive care treatment with artificial nutrition. We assumed an autoimmune process as cause of the clinical deterioration as the rapid clinical progression, the lack of systemic inflammatory signs and lack of vascular risk factors excluded exacerbated dementia, acute infectious disease, or cerebral atherosclerotic microangiopathy. An intravenous high dose therapy with methylprednisolone (1 g) for 5 days followed by a second pulse was started. The patient's vigilance improved gradually, he followed simple commands, started to answer questions and the flapping tremor disappeared. Cyclophosphamide treatment was initiated at a dosage of 750 mg/m^2^ body surface intravenously. The patient was discharged awake, disoriented, but highly dependent and unable to perform simple activities of daily life or to walk.

Since then, cyclophosphamide treatment has been repeated monthly, leading to a striking clinical improvement: the patient is now fully orientated, shows no tremor or other neurological deficits and is able to walk for ~100 m without help. He is able to actively support in household and garden and is again well-informed in world politics. MRI control image showed a reduction of the leukoencephalopathy (Figure 1D).

We present a case of rapid progressive cerebral small vessel disease caused by autoimmune reaction. The reversibility of the leukoencephalopathy upon strong immunosuppression together with the lack of competing causes strongly supports the diagnosis of BCG-induced molecular mimicry, leading to small-vessel cerebral vasculitis. We interpreted the left middle cerebral artery territory infarction as a correlate of small vessel autoimmune vasculitis. Our patient had received intravesical BCG-instillations against urothelial carcinoma. This is a well-known adjuvant therapy with attenuated *Mycobacterium bovis* BCG causing a boosted anti-tumor response (1). Up to 5% of the patients develop serious systemic side effects with inflammation up to life threatening sepsis (2) or immunologic reactions such as myasthenia gravis (3, 4). Two recent publications presented cases of small-vessel central nervous system vasculitis caused by BCG, but in contrast to our case, these reports pointed toward an acute BCG infection as underlying cause and patients were treated by either tuberculostatic therapy only (5) or in combination with steroids (6). Based on the pronounced response to immunosuppressive treatment with cyclophosphamide, we conclude an autoimmune etiology due to previous BCG instillations. A cancer-related vasculitis is rather unlikely, considering the clinical course and the PET imaging results. Immune-mediated cerebral vasculitis should be considered in patients with rapidly progressive dementia-like symptoms treated with BCG, as an immunosuppressive treatment can be highly effective even at severe clinical stages.

## Data Availability Statement

All datasets generated for this study are included in the article/supplementary material.

## Ethics Statement

Ethical review and approval was not required for the study on human participants in accordance with the local legislation and institutional requirements. The patients/participants provided their written informed consent to participate in this study. Written informed consent was obtained from the individual(s) for the publication of any potentially identifiable images or data included in this article.

## Author Contributions

IQG, KF, and SW conceived the case report design. AB and JS collected clinical data and AB drafted the manuscript. SW, SB, and MU revised the manuscript. UY provided neuroradiological input and figures. All authors read the manuscript, provided critical feedback, and approved the final manuscript.

## Conflict of Interest

The authors declare that the research was conducted in the absence of any commercial or financial relationships that could be construed as a potential conflict of interest.

## Abbreviations

BCG: Bacille Calmette-Guérin
MRI: magnetic resonance imaging
CSF: cerebrospinal fluid
EEG: electroencephalogram
PET: positron emission tomography.
